# Investigating Plasma‐Activated Water (PAW) on *Aspergillus flavus* in Almonds and the Study of Its Effect on the GPI Receptor of the Fungus

**DOI:** 10.1002/fsn3.70188

**Published:** 2025-04-21

**Authors:** Mohammad Javad Aarabi, Sajad Rostami, Bahram Hosseinzadeh Samani

**Affiliations:** ^1^ Department of Mechanical Engineering of Biosystem Shahrekord University Shahrekord Iran

**Keywords:** *A. flavus* fungus, almonds, molecular dynamics, plasma‐activated water

## Abstract

Plasma‐activated water (PAW) represents an innovative application of non‐thermal plasma technology that can potentially enhance water treatment processes. A recent study investigated the efficacy of plasma‐activated water in inhibiting *Aspergillus flavus* in almonds. The Study demonstrated that the duration of plasma‐activated water treatment most significantly diminished fungal presence, followed by water flow rate and the argon‐to‐combined gas ratio. Plasma‐activated water eliminated 2.6 logarithmic units of fungi across multiple experimental regimens. Plasma diminished water flow, further reducing *A. flavus* fungus. Extending activated water application from 1 to 10 min diminished *A. flavus* by 1.17 logs. The research revealed that ^1^O_2_, NO_3_−, and H_2_O_2_ influenced the GPI receptor in distinct manners. The quantity of hydrogen bonds between molecular GPI and the solvent in H_2_O_2_ exhibited a wider variety of effects than the two free radicals. The Study indicates that plasma‐activated water eradicates *A. flavus* fungus by targeting the GPI receptor.

## Graphical abstract

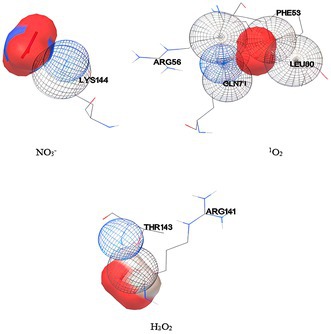

## 1 Introduction

The spread of diseases caused by contaminated food is a significant issue that has arisen since harvesting agricultural products and food industries (Hazards 2013). One frequent issue in agricultural products involves contamination with aflatoxin (Toyofuku et al. 2020). Aflatoxin threatens food safety and restricts the trade of dried fruits like walnuts, almonds, and pistachios (Campbell et al. 2003). Considering the position of almonds in Iran's economy, the factors threatening the market of this product should be carefully investigated and explained (Ziaolhagh 2011). Controlling aflatoxin contamination is crucial for ensuring the safety of almonds for consumption and trade (Whitaker et al. 2010). *A. flavus* is a significant source of aflatoxin in dried fruits like almonds (Tasouji 2018; Venkatesh and Keller 2019). Researchers have investigated new technologies to solve the problem of contamination of agricultural products with *A. flavus* fungus, and non‐thermal plasma is one of these methods (Guo et al. 2021). Using non‐thermal plasma to cover the heterogeneous surface of agricultural products has limitations in performance. Therefore, researchers have investigated plasma‐activated water (PAW) as an alternative to overcome this problem (Samukawa et al. 2012).

It has been found that PAW can control bacteria, microbes, and fungi and act directly on plants and agricultural products (Yang 2019). According to research on PAW, reactive oxygen species (ROS) and reactive nitrogen species (RNS) are the primary active ingredients. Hydroxyl radicals, hydrogen peroxide, singlet oxygen, superoxide anions, and ozone are the primary constituents of ROS. In contrast, nitrate, nitrite, peroxynitrite, nitric oxide radical, ammonia, and nitrogen are the primary constituents of RNS (Guo et al. 2021). The presence of radical active species in plasma‐activated water is a factor that affects the life of bacteria, microbes, and fungi and controls their reproduction and survival. One of the important factors in the survival and reproduction of fungi is the connection of protein chains from the cell surface to the cell nucleus. In fungi, this activity is in charge of surface receptors. One of the most important receptors in fungi is the GPI anchor. The GPI (Glycosylphosphatidylinositol) receptor makes it possible for proteins to be attached to cells, thus maintaining the fungus's cell structure (Karplus and McCammon 2002). This connection determines the correct position of proteins in the cell structure, and in this way, it is effective in the optimal functioning of the cell, communication with the external environment, and response to environmental conditions.

For this reason, the destruction of the GPI anchor can disrupt the cell structure, reduce its stability, and directly or indirectly lead to the violation of the biological activities of the fungus (Menon 2013). Molecular dynamics modeling can be performed to examine the effects and mechanisms of plasma‐activated water, the active species it generates, and the GPI receptor's critical role in fungi growth and reproduction. Molecular dynamics (MD) simulations forecast the movement of individual atoms inside a protein or other molecular system over time due to interatomic interactions (Karplus and McCammon 2002).

The fundamental and straightforward application of simulation is the assessment of the mobility or flexibility of various components of a biological molecule. This Study evaluated the impact of plasma‐generated activated water on eradicating *A. flavus* fungus in a laboratory setting, employing a molecular dynamics simulation method to examine its efficacy against the fungus. The impact of various active species, including nitrate (NO_3_^−^), hydrogen peroxide (H_2_O_2_), and singlet oxygen (^1^O_2_), on the inhibition or alteration of the GPI receptor was examined by molecular dynamics simulation.

## 2 Materials and Methods

### 2.1 Methodology for Plasma‐Activated Water Production

**FIGURE 1:**
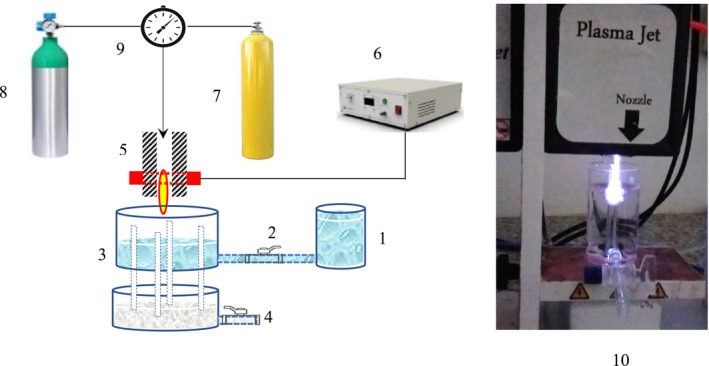
Plasma‐activated water system: (1) water inlet tank, (2) control valve, (3) treatment tank with a diameter, (4) flow outlet pipe that is built into the treatment tank, and the plasma‐treated water comes out of it, (5) assembly DBD plasma and plasma spray, (6) plasma generator, (7) Argon tank, (8) air tank, (9) gas volume ratio regulator and (10) inset photo of the actual system.

Considering the need to apply plasma to water to create PAW, an atmospheric pressure cold plasma jet with a dielectric barrier discharge method has been used to produce plasma, and the system was designed in the Study (Esmaeili et al. 2023). In the system designed by applying plasma to continuous flow, plasma‐activated water will be produced (Figure 1).

In the treatment container, the incoming water will be slowly affected by the plasma and continuously exit from the outlet. Using a one‐way valve will allow control of the flow entering the treatment tank. In the testing conditions, the input and output flow will be controlled and adjusted until the desired stable flow is reached so that the appropriate time of plasma treatment occurs for the water and there is no interruption in the continuity of the flow (Figure 1).

Plasma‐activated water is created using deionized water, different combinations of argon gas and air, and flow rates of 0.5, 1, and 1.5 mL/min for the water flow through the system. In this way, plasma‐activated water is prepared for application on *A. flavus* fungus, and its effect on the control of the fungus will be studied with the duration of application of 1, 10, and 20 min on the samples.

### 2.2 Effect of Plasma‐Activated Water on *A. flavus*

This Study utilized the *A. flavus* fungus (PTCC no. 5004), which was procured from the Iran Scientific and Industrial Research Organization. Fungi were cultivated on Potato Dextrose Agar (PDA) for 5 days at 28°C. Fungi cultivated in the laboratory were utilized as fungal stock. After 5 days, the plates were preserved at 4°C for additional examination (Devi et al. 2017).

### 2.3 Product Exposure to *A. flavus* Fungus

The dry and raw almonds used in this research were of the Ma'mai variety, obtained from Saman City, Chaharmahal, and Bakhtiari province. The prepared almonds were kept at room temperature and humidity in closed nylon bags from the time of preparation until the time of testing. To inoculate the fungus on almonds, first, the samples were disinfected by UV radiation to prevent the possible growth of other microorganisms. The colony count influences the test outcome, perhaps leading to inaccuracies due to the presence of other fungi. The samples were immersed in 70% ethanol and subsequently rinsed with distilled water. Following the extraction of spores from the growth medium, they were suspended in sterile distilled water with a concentration of one‐tenth percent Tween 80, resulting in a homogeneous suspension of fungal spores.

The spores in the suspension were calibrated utilizing the half‐McFarland technique. A total of 15 petri dishes were created, with 10 almond kernels allocated to each dish. Subsequently, 2 μL of the resultant spore mixture were injected onto the surface of the samples using a sampler and allowed to dry under a laminar flow hood for 1 h (Devi et al. 2017).

### 2.4 Mycological Test After Applying Plasma

Subsequent to plasma application, the almond samples were maintained in a tube containing 8.9 mL of 10% peptone water at ambient laboratory temperature for a duration of 2 h. Subsequently, dilutions of 1:50, 1:500, 1:5000, and 1:50000 of the sample were produced in tubes containing 9 mL of 10% peptone water. From each dilution, 100 μL were inoculated into the PDA culture medium and incubated in a greenhouse at 28°C for several days. All test procedures were conducted under a laminar flow hood. Cultural media were examined daily. Upon counting the colonies, the test findings were quantified as the number of CFU (colony‐forming units) of fungi in each sample, and the subsequent relationship was employed to articulate the reduction in colony numbers across several treatments.

(1) $$\mathrm{Log}\;{\text{Reduction}}_{=}\;\mathrm{Log}\;\left(\frac{n_{0}}{n}\right)$$

Where n_0_ is the number of colonies before treatment with plasma‐activated water and n is the number of colonies after treatment with plasma‐activated water (Devi et al. 2017).

### 2.5 Process of Analyzing, Modeling, and Optimizing Experimental Data

By using the response surface method, the effect of variable indicators in the plasma‐activated water production system, including the flow rate of water under plasma treatment, the ratio of argon to the combined plasma generator gas, and the duration of applying plasma‐activated water to the samples on the amount of *A. flavus* fungus reduction was identified in almond samples. The optimal performance mode of plasma‐activated water was determined to reduce *A. flavus* fungus in almonds using the obtained data and their analysis. The response level method determines the relationship between a response and independent variables, which includes the following three steps (Hosseinzadeh Samani et al. 2020).

The initial step involves identifying independent variables, testing levels, and the test plan type; the subsequent step entails forecasting and examining the model equation's accuracy, and the final step consists of generating response curves and a linear representation of the response relative to independent variables. The resolution of the subsequent equation was utilized 15 times to achieve the ideal value.

(2) $$Y_{i}=\beta_{0}+\sum {Β}_{i}\;X_{i}+\sum \beta_{\mathrm{ii}}\;{X_{i}}^{2}+\sum \beta_{\mathrm{ii}}\;X_{i}\;X_{\mathrm{ij}}+\varepsilon$$

In which *β*_o_, *β*_i_, and *β*_ii_ are constant coefficients, *X*_i_ and *X*_ij_ are independent variables in the process, and ε is a random error; according to the available sources, the range of changes of independent variables in the experiment was selected according to Table 1, and the amount of *A. flavus* fungus reduction was the dependent variable The test was determined (Hosseinzadeh Samani et al. 2020).

**TABLE 1:** Levels of independent variables selected in response level method for *Aspergillus flavus* fungus.

Independent variables	Coding levels		
	−1	0	1
Water flow rate (mL/min)	0.5	1	1.5
The ratio of argon	0	0.5	1
Time to apply activated water	1	10	20

### 2.6 Computational Phase Analysis Using Molecular Dynamics Simulation

The alterations induced by plasma‐activated water on the GPI receptor were examined by an in silico molecular dynamics simulation to assess the impact of plasma‐activated water on *A. flavus* fungus. Consequently, to create the input file for the Gromacs software simulation, it was essential first to compile the beginning file, including the specified radicals at a defined concentration in an aqueous medium and the *presence of A. flavus fungus*. The portable version of the Packmol program was employed to arrange each of the three analyzed free radicals—H_2_O_2_, ^1^O_2_, and NO_3_^−^—at their ideal concentrations, as determined by experiments, within a box holding water and fungus. The output of each file was generated and preserved in PDB format (Justino et al. 2021). Calculations related to the data output from Gromacs, by Excel 2016 software, were analyzed, and the results were presented in the form of figures and tables with mean ± SD values.

To prepare the simulation conditions of activated water with plasma, firstly, the structure of several radical active species such as nitrate (NO_3_−), hydrogen peroxide (H_2_O_2_), and singlet oxygen (^1^O_2_) from the PubChem website and respectively with the codes 943, 784, and 977 were received in SDF format. Then, due to the absence of the three‐dimensional structure of the mentioned compounds in the database, the three‐dimensional structure of these compounds was drawn using Chem 3D version 18.0 software. Also, the third structure of GPI with code 64173771 was received from Pubchem (Farhadian et al. 2022). Before starting the simulation, the energy minimization of the compounds was done with chimera ucsf 1.13.1 software, and the output of each file was saved in pdb format. A molecular dynamics simulation was done by creating a separate file for each free radical using GROMACS version 5.1.4 software. Since the target structures of this study did not have any of the four structures of protein, nucleic acid, lipid, and sugar that GROMACS can identify, the structures were prepared through the Acpype server to create topology files (https://www.bio2byte.be/acpype). This study selected the Amber force field. Then, the three‐dimensional file of each structure was introduced to the GROMACS software, and the coordinate file (gro) was created using the edition command. Also, in the simulation's implementation, the simulation's duration was defined as 40 ns (40,000 ps).

The pdb structure of the free radical complex and GPI anchor at the position with the least distance was obtained to investigate the interaction of free radicals created by activated water with plasma and GPI receptor composition in *A. flavus* fungus. For this purpose, Autodesk 4.2 software was used in such a way that the three‐dimensional structure of the three free radicals investigated was obtained with Arguslab software in pdb format, and the three‐dimensional structure of the GPI anchor receptor was obtained from the protein database at www.rcsb.org. It was downloaded, and then their spatial position was observed with PYMOL visual software (Scudellari 2020).

## 3 Results and Discussion

Design Expert software, response surface curve method, and Box–Behnken design were used to determine the experimental treatments, and the results of all 15 experiments with three repetitions are mentioned in the central point. The reduction in *Aspergillus fungus* at different times is given in Table 2.

**TABLE 2:** The amount of *Aspergillus flavus* fungus reduction at different times.

Run	A: Q (mL/min)	B: Ar%	C: T_w_ (min)	*Aspergilus* log reduction
1	1.00	0.50	10.50	−2.18
2	0.50	0.50	1.00	−0.97
3	1.50	0.50	20.00	−1.78
4	0.50	0.00	10.50	−2.1
5	1.00	0.00	20.00	−2.28
6	1.00	1.00	1.00	−1
7	0.50	1.00	10.50	−2.22
8	1.00	0.50	10.50	−2.15
9	0.50	0.50	20.00	−2.4
10	1.50	1.00	10.50	−1.7
11	1.00	0.00	1.00	−1
12	1.00	0.50	10.50	−2.14
13	1.50	0.00	10.50	−1.7
14	1.50	0.50	1.00	−0.63
15	1.00	1.00	20.00	−2.3

### 3.1 The Results of Statistical Analysis and Data Modeling

In this modeling, the reduction of *A. flavus* fungus was considered as a dependent variable, and the variables used in the process, including the flow rate of water under the influence of plasma [Q (mL/min)], the ratio of argon to the combined gas [Ar (%)], and the time of application of activated water [T_w_ (min)] as independent variables in were considered The results of the analysis of variance of the main and mutual effects of the three factors Q, Ar, and T_w_ (min) showed that it has a significant effect at the 10% level on the reduction of *A. flavus* fungus in almond products. According to the statistical analysis results in Table 3, the significance coefficient (*p* value) less than 0.1 indicates the significance of the model factors. Also, the significance of the model and the non‐significance of the lack of fit indicate that the model has sufficient accuracy. All parameters examined in this Study except B, (BC), and (B2) were significant at the 10% level.

**TABLE 3:** Results of statistical analysis of test data.

Source	Sum of squares	df	Mean square	*F*‐value	*p* value
Model	4.83	7	0.6901	1572.02	< 0.0001
A‐Q	0.4418	1	0.4418	1006.35	< 0.0001
B‐Ar	0.0025	1	0.0025	5.58	0.0502
C‐T_w_	3.33	1	3.33	7581.13	< 0.0001
AB	0.0036	1	0.0036	8.20	0.0242
AC	0.0196	1	0.0196	44.65	0.0003
A^2^	0.1674	1	0.1674	381.36	< 0.0001
C^2^	0.9186	1	0.9186	2092.43	< 0.0001
Residual	0.0031	7	0.0004		
Lack of Fit	0.0022	5	0.0004	1.02	0.5632
Pure Error	0.0009	2	0.0004		
Cor Total	4.83	14			

**FIGURE 2:**
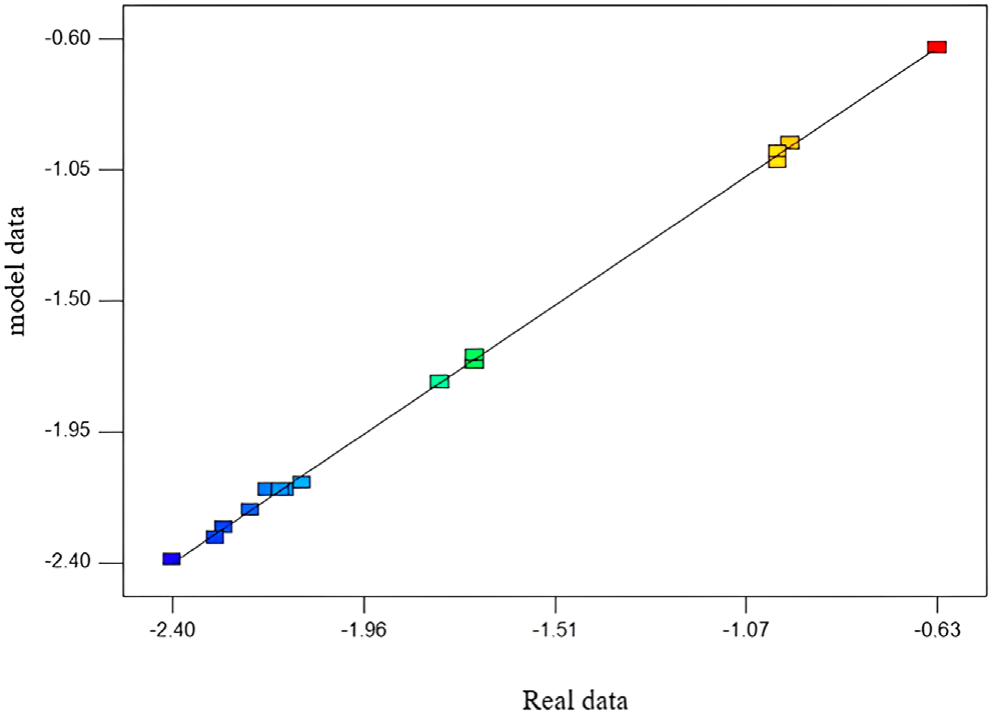
Comparison of real data and model data.

Figure 2 shows a comparison between actual data and model data. According to this graph and the correspondence and closeness of the model data to the actual data, it was found that there is a very good correlation between the results obtained by the experimental method and the values predicted by the statistical methods.

Equation (3) was extracted from the data analysis. The coefficient of explanation, standard error, and coefficient of variation (CV) were obtained as 0.9994%, 0.021%, and 1.18%, respectively.

(3) $$\begin{array}{cc} \text{Aspergilus}\;\mathrm{Log}\;\text{Reduction}\;=\; & -0.211627+1.45140\;Q-0.198061T_{w}\\ & +0.12\;Q^{*}\mathrm{Ar}+0.014737\;Q^{*}T_{w}-0.001053\;{\mathrm{Ar}}^{*}\;T_{w}\\ & +0.853333Q^{2}+0.053333{\mathrm{Ar}}^{2}+0.005522\;{T_{w}}^{2} \end{array}$$

Non‐significant coefficients that include the ratio of argon to the Ar* T_w_ and the Ar^2^ from the relationship (3) were removed, and the final coded relationship was extracted (4).

(4) $$\begin{array}{cc} \text{Aspergillus}\;\log\;\text{Reduction}\;=\; & -2.15+0.2350\;Q-0.0175\;\mathrm{Ar}\\ & -0.6450\;T_{w}+0.0300\;Q^{*}\mathrm{Ar}+0.0700\;Q^{*}T_{w}\\ & +0.2123\;Q^{2}+0.4973\;{T_{w}}^{2} \end{array}$$

In Equation (4), the negative coefficient indicates the decrease of *Aspergillus flavus*. A larger negative coefficient indicates a greater reduction of *A. flavus* . According to the coded Equation (4) and the numerical values of the coefficients, it is clear that T_w_ has the greatest effect on the reduction of *A. flavus* fungus, followed by Q and Ar has the most significant effect on reducing this fungus.

### 3.2 The Effect of Some Parameters of PAW on *A. flavus*

**FIGURE 3:**
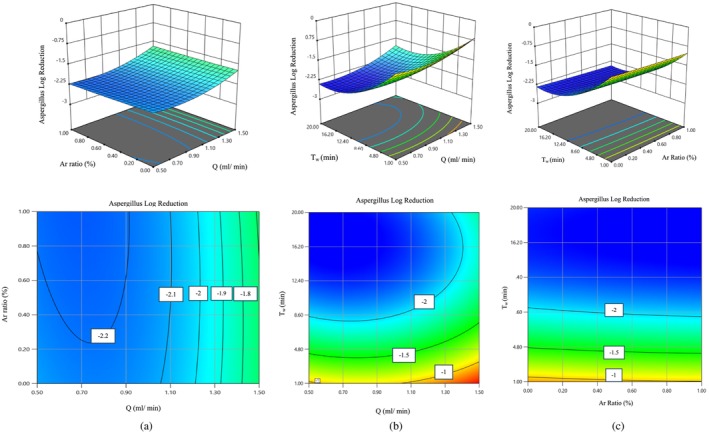
Change in logarithmic reduction of *A. flavus* by (a) 3D and contour diagram of argon ratio and water flow rate under the influence of plasma, (b) 3D and contour diagram of water flow rate under the influence of plasma and time of water application, (c) 3D and contour diagram of water flow rate under the influence of argon ratio and time of water application.

The effect of independent variables Q, Ar, and T_w_ on the amount of logarithmic reduction of *A. flavus* fungus at a central point is shown (Figure 3). Figure 3a shows the effect of independent variables Ar and Q on the logarithmic reduction of *A. flavus* fungus. As it is clear from this graph, the slope of the Q changes is greater than the slope of the graph of the Ar, so this variable has a greater effect on the logarithmic reduction of *A. flavus fungus*. Also, due to the larger negative coefficient of Q compared to the negative coefficient of the Ar, in coded relation (3), we can also draw the same conclusion. According to this diagram, it is clear that the further away from the central point, the effect of the incoming gas on the reduction of the fungus decreases. Therefore, it can be concluded that when an equal amount of argon gas and air are combined, they have the greatest effect on the reduction of *Aspergillus flavus*. *A. flavus* decreased by 0.01 log by increasing the composition ratio of incoming argon gas from 0 to 0.5, and by increasing the composition ratio from 0.5 to 1, the amount of *A. flavus* fungus decreased from −2.14 to −2.16. Due to the creation of plasma with a source of argon and air gases, highly reactive RNS and ROS species are created, which react with the surface of water or penetrate it and create complex reactions. One of the most important reasons for the effect of plasma created from different gases on *A. flavus* fungus is the creation of free radicals and highly reactive species and their dissolution and penetration in plasma‐activated water.

In such a way that the more free radicals and reactive species are present in the plasma‐activated water, the more damage is done to the fungus in the samples. The free radicals produced by applying plasma‐activated water have different concentrations according to the type of gas used, so in air, plasma‐activated water, single oxygen free radicals, nitrogen monoxide, nitrate, and nitrite are more. Compared to activated water, it is created with argon plasma (Liu et al. 2020; Llamas et al. 2006).

Also, in water activated with argon plasma, there are more free radicals of positive single hydrogen, hydroxyl, and hydrogen peroxide than in water activated with air plasma (Liu et al. 2020; Llamas et al. 2006). They produce free radicals. In this way, a suitable concentration of RNS and ROS penetrates the water activated with plasma, and applying it to the samples destroys the structure of the *A. flavus* fungus and thus controls the fungus in the samples.

The increase in the Q leads to a decrease in the time of water's effect on plasma, and the decrease in flow will lead to an increase in the time of water's effect on plasma. According to this diagram, by increasing the duration of plasma application from 1 to 5.5 min, the Q, the logarithmic reduction rate of *A. flavus* fungus reaches from −1.7 to −2.14, and by increasing the time to 10 min, this trend A decrease reaches 2.17.

Therefore, it can be concluded that by increasing the duration of plasma application on water, free radicals, positive and negative ions, highly reactive ROS, and RNS species will be created and penetrate the water and will increase. With the increase of these highly reactive species, water's reducing potential and oxidizing properties will increase. With the decrease in pH, the acidity of the environment will increase. Treatment of almond samples infected with *A. flavus* fungus with water activated with plasma leads to the destruction of the fungus and its reduction.

Figure 3b,c shows the reduction of *A. flavus* fungus under the Q and T_w_. It also shows the ratio of argon gas to the gas composition entering the plasma system and the time of plasma‐activated water application on the samples. According to the slope of the graphs and the coefficients of the coded relationship (3), it is clear that the T_w_ has a greater effect on the reduction of *A. flavus* than the Q.

According to this diagram and what was said before, with the increase of Q, the logarithmic reduction of *A. flavus* fungus decreases. Also, according to this diagram, the longer the duration of the activated water application compared to the central point, the greater the reduction of *A. flavus* . By increasing the T_w_ from 1 to 10.5 min, *A. flavus* decreases from a log cycle of −1 to −2.14. Also, with time up to 20 min, the logarithmic decrease of *A. flavus* to −2.30 arrives. The longer the activated water is applied to the samples, the more opportunities for reaction and destruction are available to ions and free radicals. In such a way, there is more opportunity for the oxidation of structures and the effect of free radicals on *Aspergillus* fungus. Therefore, by increasing the duration of water application to the samples, the fungus control is better, and the fungal colonies are reduced.

In a similar research conducted by Yao et al. (2023), the fungicidal ability of plasma‐activated water has been proven. Also, the results of their research showed that water activated with plasma has the best efficiency in eliminating *A. flavus* fungus. After treatment for 60 min, this water reduced the logarithm of *A. flavus* by 22.3 and damaged the integrity of this fungus's cell membrane.

**FIGURE 4:**
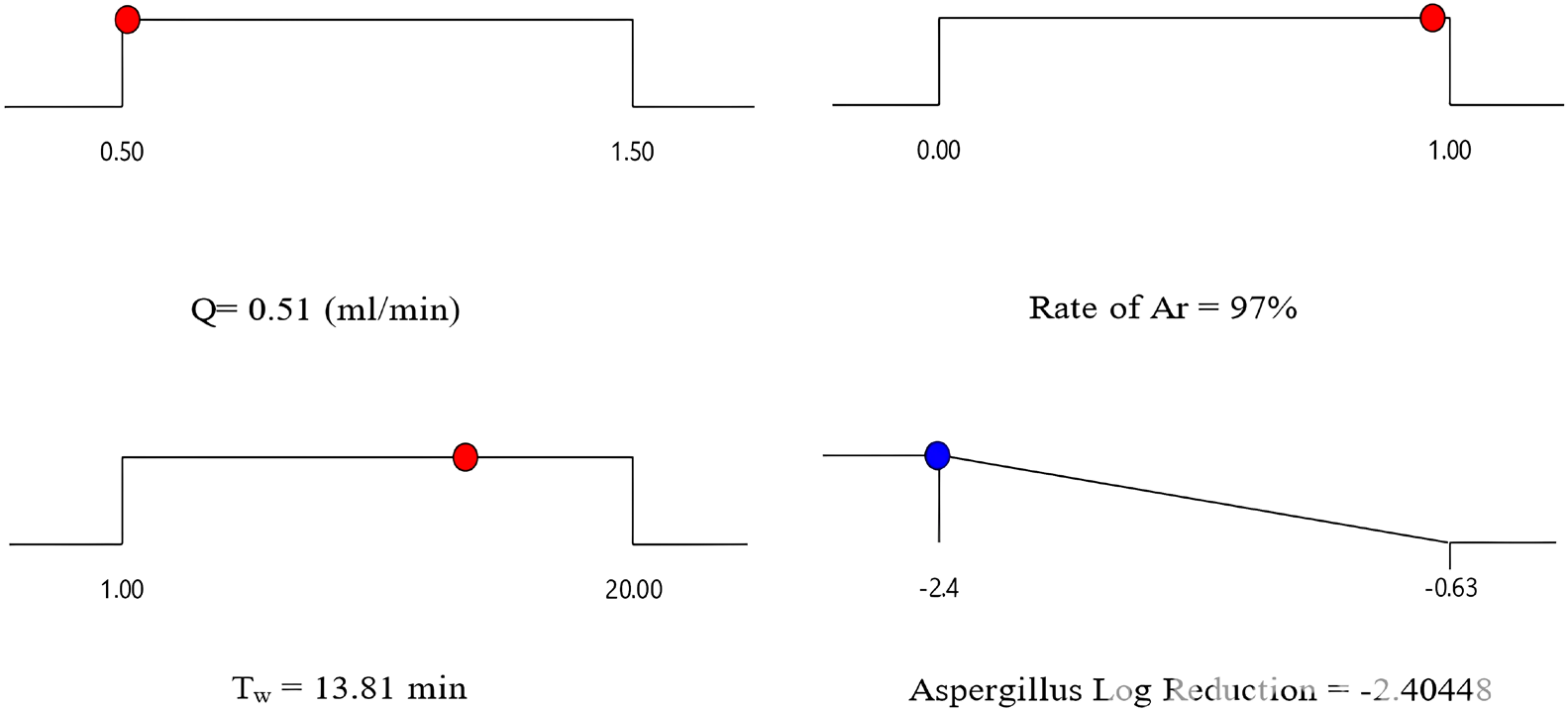
Process optimization based on response surface model for plasma‐activated water system.

The results of the optimization of the response surface method with the greatest reduction of *A. flavus* are given in Figure 4. The optimal values in the designed system and available parameters for the production and application of plasma‐activated water for Q, Ar, and T_w_, respectively, 0.51 mL/min, 97% Argon, and 13.81 min, were obtained. For the obtained values, 2.4 logarithmic units have been reported to reduce *Aspergillus flavus*. The reduction rate of *Aspergillus fungus* was investigated in a laboratory under these conditions to validate the optimal points obtained in the response surface method. At this point, the amount of fungus reduction was 2.48 logarithmic units, which showed that the theoretical and laboratory optimum had a small difference, showing the optimization method's accuracy.

### 3.3 The Results of In Silico Molecular Dynamics and Laboratory Simulations

The data obtained in the optimal conditions of the experiments were used to investigate the effect of plasma‐activated water on the structure of *A. flavus* fungus. The concentration of three target free radicals was measured Using a plasma‐activated water production system in optimal conditions (97% argon gas and water flow rate 0.51 mL/min).

The measured concentrations were about 4 ppm for H_2_O_2_, 22 ppm for ^1^O_2_, and 5 ppm for NO_3_^−^. This concentration of free radicals is placed in a box of water and fungus, and a simulation of activated water with plasma containing (NO_3_^−^), (H_2_O_2_), and (^1^O_2_) in the presence of GPI receptor took 40 ns.

**FIGURE 5:**
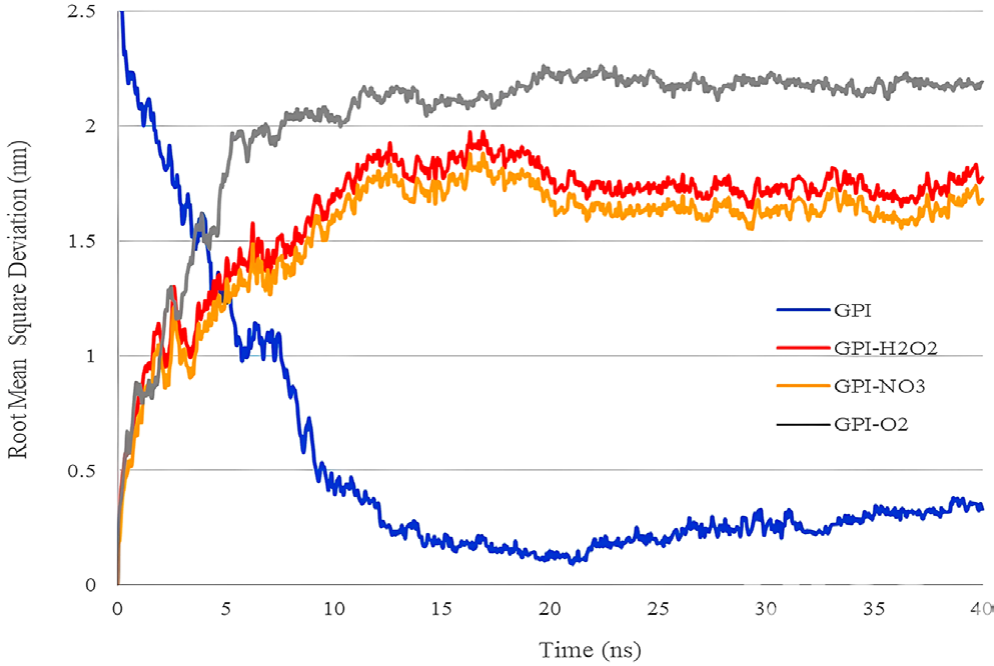
Variations (RMSD) between particles of the fungus GPI receptor in 40 ns of simulation in the presence of plasma‐activated water.

Figure 5 shows the changes in RMSD interactions between GPI receptor particles for 40 ns of molecular dynamics simulation in water. The RMSD factor indicates the changes related to the tertiary structure of the protein in the simulation and can also indicate the degree of charge of the protein (Monhemi et al. 2015). The increase in the amount of RMSD under treatments that cause changes in the composition indicates the increase in the instability of the composition in the presence of the mentioned factors (Sadeghi‐Kaji et al. 2019; Zhang and Lazim 2017).

According to the Figure, it is clear that due to the application of free radicals, the RMSD of the GPI receptor has increased. Among the applied free radicals, ^1^O_2_ produced more RMSD than the others. Regarding RMSD changes, it can be seen that H_2_O_2_ performed better in destroying the GPI receptor than NO_3_^−^. This shows that ^1^O_2_ causes more destruction and instability in the GPI receptor structure among the studied free radicals and affects this receptor more than other studied radicals.

Table 4 shows the mean and standard deviation of RMSD, RMSF, and Rg related to the composition after 40 ns of simulation without the application of plasma‐activated water and with the application of plasma‐activated water. According to the table data, the average RMSD of three free radicals ^1^O_2_, NO_3_,^−^ and H_2_O_2_ is 2.0162 ± 0.3695, 1.5449 ± 0.2805, and 1.6377 ± 0.2827, respectively. These results indicate that ^1^O_2_ causes more changes in the RMSD of the compound than the other two applied radicals.

**TABLE 4:** Mean and standard deviation of RMSD, RMSF, Rg (after 40 ns).

System	Rg (nm)	RMSF (nm)	RMSD (nm)
NO_3_^−^	1.829 ± 0.2109	0.566 ± 0.297	1.545 ± 0.2805
H_2_O_2_	2.33 ± 0.21	0.567 ± 0.298	1.6377 ± 0.2827
^1^O_2_	2.329 ± 0.2109	0.481 ± 0.3309	2.0162 ± 0.3695
(Without application of PAW)	2.196 ± 0.25	0.5119 ± 0.41	0.518 ± 0.5576

**FIGURE 6:**
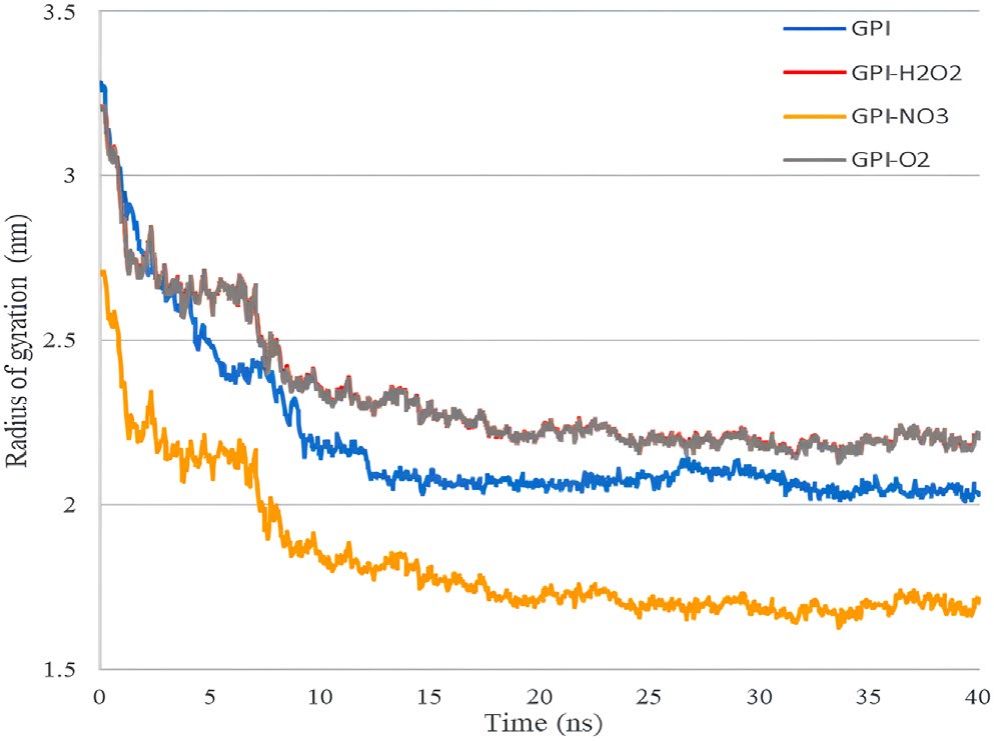
The radius of gyration or (Rg) of the fungus GPI receptor in 40 ns of simulation in the presence of plasma‐activated water.

Figure 6 shows the changes in the radius of gyration (Rg) for GPI in 40 ns of molecular dynamics simulation in the presence of plasma‐activated water. The Rg is used to evaluate the degree of compression in proteins and compounds (Mendichi et al. 2003). The radius of gyration of a molecule is the root mean square of the distance of all atoms from the center of gravity of the molecule (Farhadian et al. 2022). If a compound has a stable structure, it maintains its Rg level. This is even though if a compound is affected by a treatment and the effect of the treatment causes the compound to open and break, the amount of Rg increases during the simulation process (McGibbon et al. 2015). The smaller the radius of rotation, the more compact the desired composition will be, and finally, the possibility of its thermal stability will be higher (Bhat et al. 2020).

Based on the mentioned Figure as well as the data in Table 4, it is clear that the changes in the rotation radius of the GPI receptor in the presence of H_2_O_2_ and ^1^O_2_ are close to each other and are more significant than the initial state of GPI (without the application of free radicals). This indicates that applying these two free radicals causes an increase in the radius of rotation of GPI and thus causes instability in the structure of GPI. For the application of NO_3_^−^ free radical, it can be seen that the rotation radius of the system has decreased, which means that the application of this radical has made the GPI receptor more compact.

**FIGURE 7:**
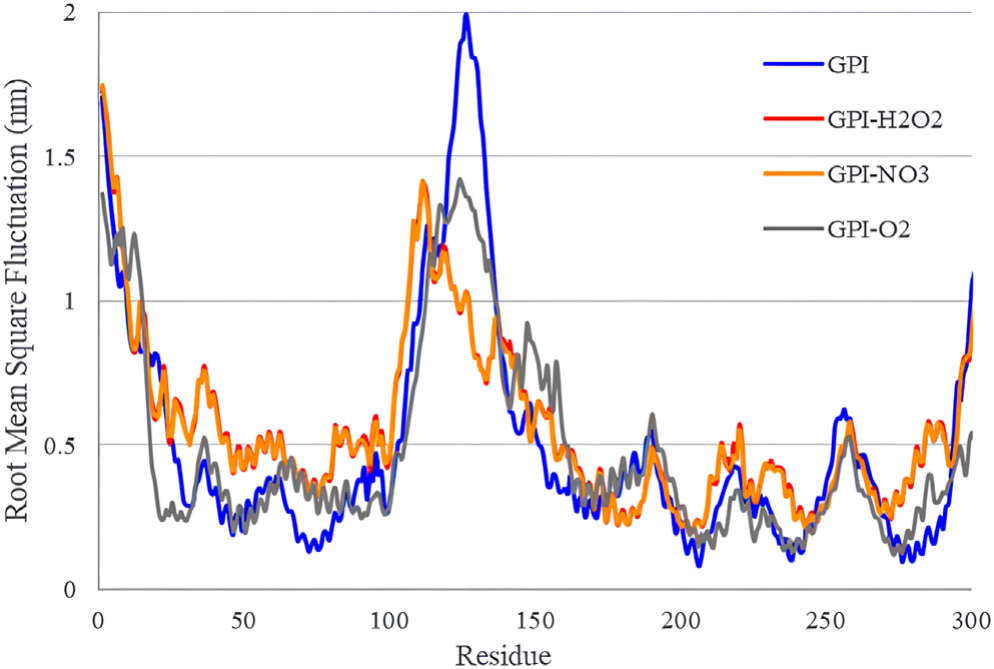
Changes (RMSF) between particles of fung GPI receptor in 40 ns of simulation time in the presence of plasma‐activated water.

Figure 7 shows the changes related to RMSF of GPI receptor amino acid units in 40 ns of simulation in the presence of plasma‐activated water. When a dynamic system fluctuates, the amount of these fluctuations can be calculated. One of the most widely used methods to calculate fluctuations is measuring the RMSF index, a key measure of the flexibility of the residues during simulation time (Ceruso et al. 1999). RMSF analysis calculates the degree of flexibility of polypeptide chains and expresses the displacement of alpha carbon atoms according to time in the position of each residue. RMSF determines the degree of flexibility of each residue or atom during the simulation time compared to the start time of the simulation of that amino acid or atom.

In each protein, the loops have the highest amount of RMSF; therefore, it is possible to examine the change in the flexibility of the loops in case of mutation or other such events (Farhadian et al. 2022). If the protein is unfolded, the amount of RMSF and entropy of the residues in them increases, while the decrease of this factor indicates stability at a higher level. Based on the Figure and the data in Table 4, it is clear that the RMSF changes of the GPI receiver for H_2_O_2_ and NO_3_^−^ are close to each other, and for ^1^O_2_ it has the lowest value. This shows that in terms of the RMSF index, ^1^O_2_ radical has the lowest effect on the corresponding receptor.

Table 5 shows the average number of hydrogen bonds between GPI receptor molecules, GPI receptors, and water as a solvent. The results show the number of hydrogen bonds for the system's state (before treatment with free radicals) and after the application of each free radical. The results show that free radicals created by plasma‐activated water have changed the number of hydrogen bonds between receptor molecules, receptor molecules, and solvents.

**TABLE 5:** The average number of hydrogen bonds in 40 ns of simulation.

System	Number of hydrogen bonds	
	GPI–GPI	GPI‐solvent
NO_3_^−^	129.57 ± 5.66	582.38 ± 20.68
H_2_O_2_	178.78 ± 8.8	592.38 ± 20.68
^1^O_2_	194.27 ± 14.53	346.305 ± 64.56
(without application of PAW)	197.27 ± 12.86	407.52 ± 34.99

**FIGURE 8:**
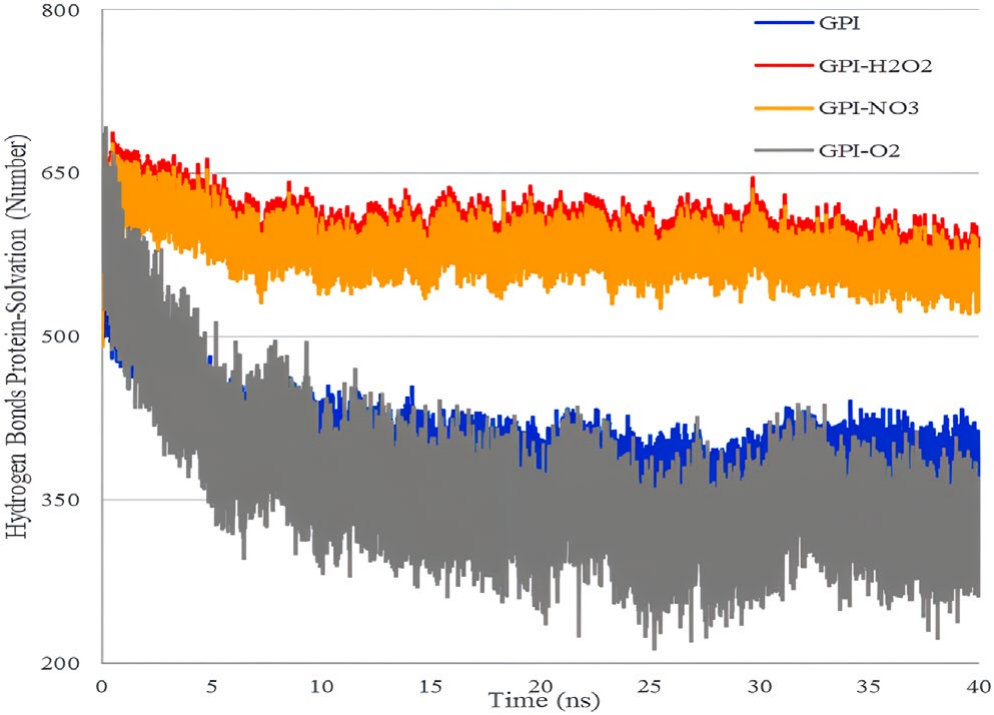
Hydrogen bond interactions of fungus GPI receptor and solvent molecules with free radicals in 40 ns of simulation.

Figure 8 shows the hydrogen bond interactions of the receptor and water solvent molecules in 40 ns of simulation. As shown in the figure, among the studied free radicals, H_2_O_2_ and NO_3_^−^ have almost the same performance in the number of hydrogen bonds, and the performance of ^1^O_2_ in this index is weaker than that of the other two radicals. The increase in the number of hydrogen bonds causes the strength of this bond to increase from the van der Waals interaction energy, and therefore, the solubility of the compound increases (Doss et al. 2012; Momeni et al. 2018).

It can be said that due to the changes in the hydrogen bond between the GPI receptor molecules and water, the solubility of the molecule has increased, and the probability that more instability will occur in its structure is higher (Ranjbar 2018).

**FIGURE 9:**
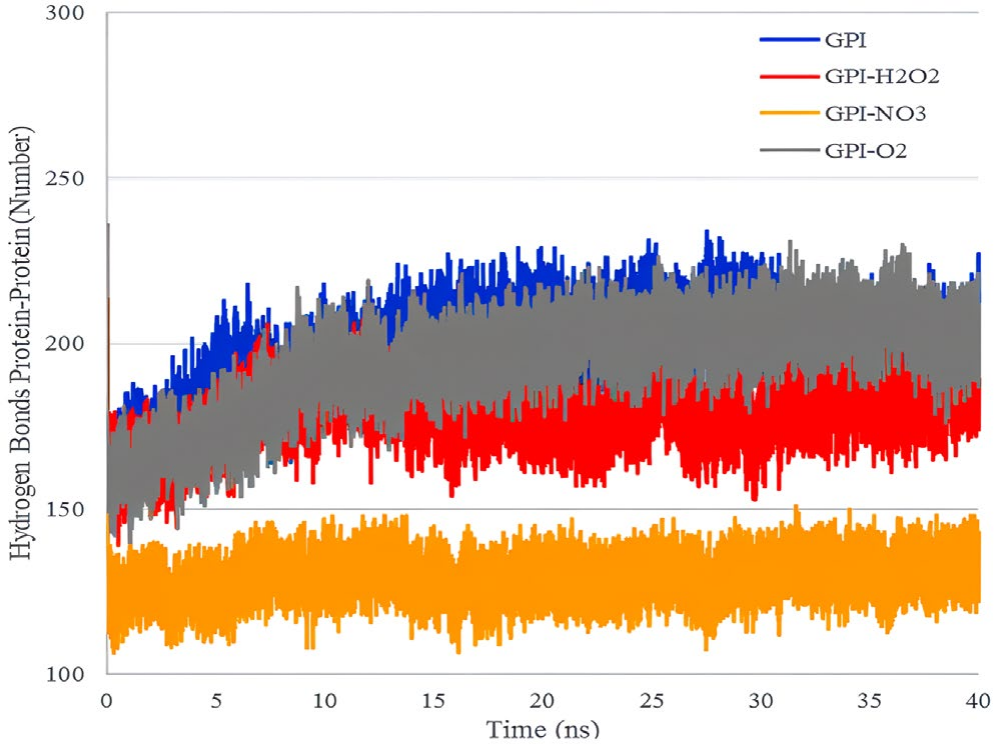
Intermolecular hydrogen bond interactions in the fungus GPI receptor, with free radicals in 40 ns of simulation.

Also, based on the data in Table 5 and Figure 9 about the number of hydrogen bonds between water and GPI, and the number of hydrogen bonds created between GPI and water (solvent), it is clear that the free radical H_2_O_2_ compared to two radicals creates more hydrogen bonds and causes more changes in GPI solubility and leads this protein to more unstable structures.

Figure 9 shows the intermolecular hydrogen bond interactions in the fungus GPI receptor with free radicals in 40 ns of simulation. As can be seen from the figure, it can be said that ^1^O_2_ has created more hydrogen bonds between the receptor molecules compared to the other two compounds. Among the three selected free radicals, the application of NO_3_^−^ caused the greatest decrease in the number of hydrogen bonds between receptor molecules. The change in the number of hydrogen bonds is a factor that shows that the applied free radicals cause changes in the protein structure of the studied receptor (Ren et al. 2017).

Table 6 shows the binding free energy between the GPI receptor and applied free radicals. Based on the data in this Table, the binding energy between the active species NO_3_^−^ and the GPI receptor has the most suitable value, and then the binding energies of H_2_O_2_ and ^1^O_2_ are close to the same value. According to the data obtained for the binding energy between GPI and free radicals, it can be stated that in comparing the three selected free radicals, NO_3_^−^ can overcome the other two radicals through higher binding to the fungus receptor compound. It has a greater impact on it and eventually changes its activity or destroys its structure (Hashemi‐Shahraki et al. 2021).

**TABLE 6:** Total binding free energy between GPI receptor and free radicals.

System	(kcal/mol) ∆G bind
^1^O_2_	−2.6 ± 0.36
H_2_O_2_	−2.36 ± 0.2
NO_3_^−^	−3.65 ± 0.4

The study provides Tables 7 and 8, which show the average energy during 40 ns of simulation, average kinetic energy, total energy over time, average temperature, and density of the system without PAW and in the presence of radicals from PAW.

**TABLE 7:** Amount of total, potential, kinetic, temperature, and density energies for the system without applying free radicals.

	System (without application of PAW)
Potential energy (kJ/mol)	−3973085.406 ± 2195.2193
Kinetic energy (kJ/mol)	720332.2865 ± 2630.6765
Total energy (kJ/mol)	−3252753.121 ± 3708.52675
Temperature (K)	300 ± 1.09563
Density (kg/m^3^)	981.3172 ± 0.9
Enthalpy (kJ/mol)	−3253966.95 ± 1658.23

**TABLE 8:** Amount of total, potential, kinetic, temperature, and density energies for the system with the application of free radicals.

	^1^O_2_	H_2_O_2_	NO_3_^−^
Potential energy (kJ/mol)	−6892362.249 ± 22,542	−4015210.807 ± 63763.36	−4016420.799 ± 13,214
Kinetic energy (kJ/mol)	1248160.521 ± 2891	730959.318 ± 11731.6295	731141.579 ± 2181
Total energy (kJ/mol)	5644201.731 ± 22850.95—	−3284251.951 ± 52301.098	−3285279.22 ± 13,544
Temperature (K)	300 ± 0.69497	299.918 ± 4.813	299.99 ± 0.895
Density (kg/m^3^)	978.849 ± 1.04	980.624 ± 15.517	980.84 ± 2.71
Enthalpy (kJ/mol)	−5643894.948 ± 22851.18	−3285099.12 ± 13511.4	−3285109.92 ± 13544.4

The data in Table 8, in comparison with the data in Table 7 show that all three free radicals can increase the system's energy level in the presence of the GPI receptor and, therefore, affect it. The obtained data shows that the ^1^O_2_ free radical has the greatest increase in the system's energy level, and the amount of change for the other two free radicals is almost identical.

The increase in the energy of the system in the presence of free radicals indicates the effectiveness of the structure from the action of free radicals on it. In other words, the increase in system energy indicates the effect of plasma‐activated water on the GPI receptor structure (Momeni et al. 2018).

**FIGURE 10:**
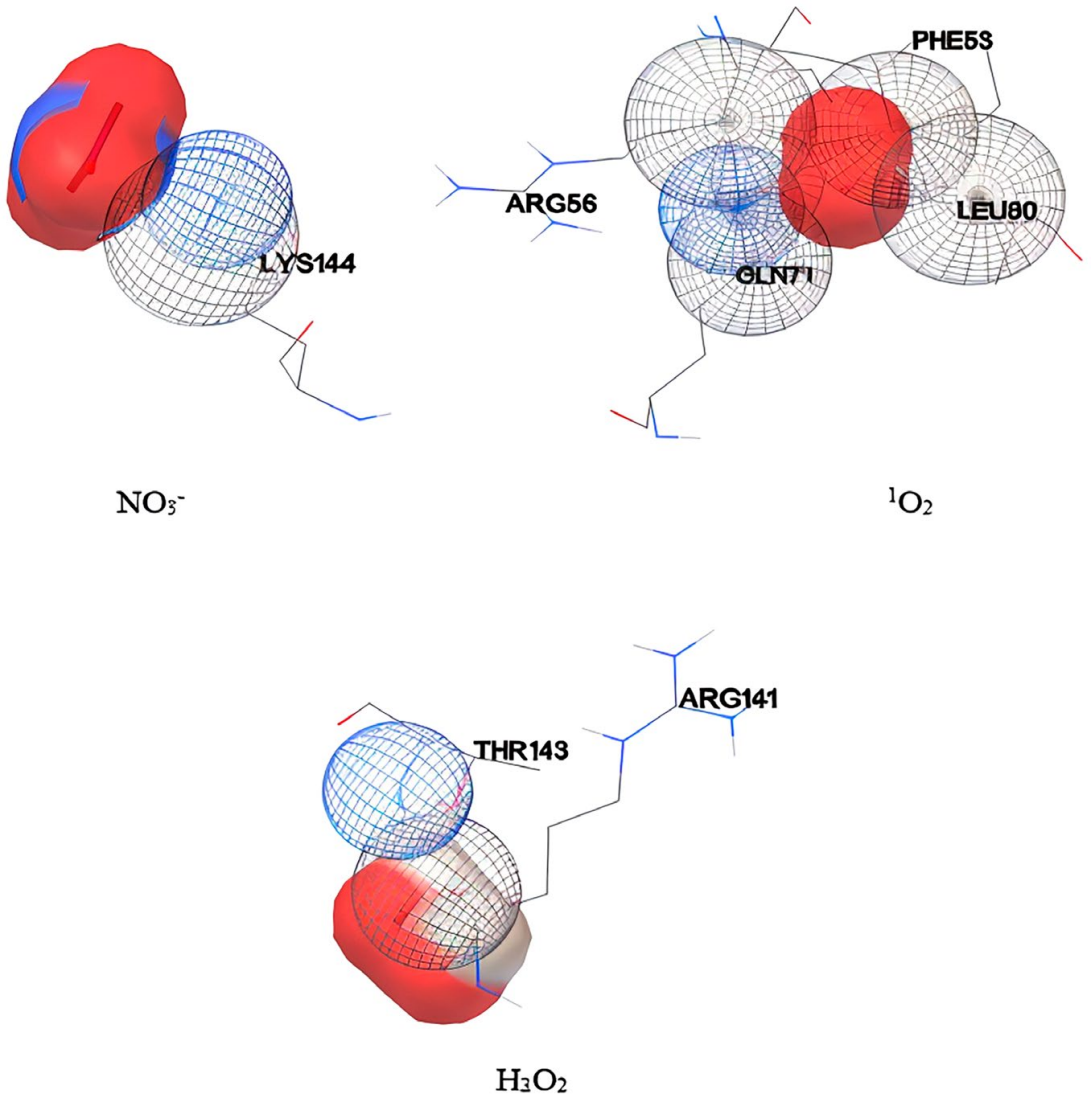
The interact simulation of the atoms involved in NO_3_−, ^1^O_2_, and H_2_O_2_ free radicals with the GPI compound in the shortest distance.

To find out how the atoms of free radicals are connected with the combination of GPI anchor receptors, the corresponding three‐dimensional structure of the complex was obtained when they have the smallest distance, and then the spatial positions and orbitals involved, which can be seen in Figure 10 is displayed. These images, obtained from the output of Autodock 4.2 software, show how free radicals interact with GPI receptor combinations in the best and closest state of interaction between free radicals and receptor combinations (Farhadian et al. 2016). As is evident in this picture, ^1^the O_2_ free radical binds with the amino acids Arginine 56 (Arg 56), Glutamine 71 (Gln 71), Leucine 60 (Leu 60), and Phenylalanine 53 (Phe 53) of GPI receptors. Also, the output images of the software show that the NO_3_^−^ free radical has bonded with the amino acid lysine 144 (Lys 144).

In the same way, the obtained images show the binding of H_2_O_2_ free radical molecules with amino acids Arginine 141 (Arg141) and Threonine 143 (Thr143) of GPI receptors. By determining how free radicals interact with the receptor structure and affect amino acids, it is clear how the compounds obtained from plasma‐activated water affect the GPI receptor structure and consequently affect *Aspergillus flavus*.

## 4 Conclusion

A study was conducted to determine the effectiveness of plasma‐activated water in controlling and removing *A. flavus* from almonds. The study found that the T_w_ had the greatest impact on reducing the *A. flavus* fungus, followed by the Q and the Ar. Different treatments were tested, and a 2.6 logarithmic unit decrease in *A. flavus* fungus in almonds was observed. Additionally, the effects of plasma‐activated water on the GPI membrane, an effective receptor in the structure of *A. flavus* fungus, were studied using the MD simulation technique under the influence of active species of free radicals (NO_3_−), (H_2_O_2_), and (^1^O2).

Based on RMSD between GPI particles when exposed to free radicals, it was found that the average RMSD of three free radicals, namely ^1^O_2_, NO_3_^−^, and H_2_O_2_, was 2.0162 ± 0.3695, 1.545.0 ± 0.2805, and 0.2827 ± 1.6377, respectively. Additionally, the gyration radius of GPI in the presence of H_2_O_2_ showed a greater impact on the receptor's structure than the other two free radicals, with a value of 2.3 ± 0.21. Moreover, the RMSF changes of the GPI receptor in the presence of H_2_O_2_ had more effects on the receptor's structure than the other two free radicals, with a value of 0.567 ± 0.298. Also, the average number of hydrogen bonds between the molecular GPI and the solvent in the presence of H_2_O_2_ had a higher effect range than the two free radicals. The research showed that plasma‐activated water can control *A. flavus* fungus in almonds. The molecular simulation showed that the free radicals in PAW can affect the structure of *A. flavus* fungus and cause its destruction by affecting the GPI receptor.

## Author Contributions

**Mohammad Javad Aarabi:** conceptualization (equal), data curation (equal), formal analysis (equal), investigation (equal), software (equal), validation (equal), writing – original draft (equal).

## Data Availability

Data available on request from the authors.
